# Outdoor light at night and neuropsychiatric symptoms in dementia

**DOI:** 10.1007/s11357-025-01745-z

**Published:** 2025-07-15

**Authors:** Manuela Tondelli, Tommaso Filippini, Giulia Vinceti, Najara Iacovino, Teresa Urbano, Sofia Costanzini, Francesca Despini, Claudia De Luca, Simona Tondelli, Marco Vinceti, Annalisa Chiari, Giovanna Zamboni

**Affiliations:** 1 Department of Biomedical, Metabolic, and Neural Sciences, University of Modena and Reggio Emilia, Modena, Italy; 2 Neurology Unit, Baggiovara Hospital, AOU Modena, Modena, Italy; 3 Department of Biomedical, Metabolic and Neural Sciences, Environmental, Genetic and Nutritional Epidemiology Research Center (CREAGEN), University of Modena and Reggio Emilia, Modena, Italy; 4 School of Public Health, University of California Berkeley, Berkeley, CA USA; 5 DIEF Department of Engineering “Enzo Ferrari”, University of Modena and Reggio Emilia, Modena, Italy; 6 Department of Architecture, Alma Mater Studiorum University of Bologna, Bologna, Italy; 7 Department of Epidemiology, Boston University School of Public Health, Boston, MA USA

**Keywords:** Dementia, Light at night, Neuropsychiatric inventory, Neuropsychiatry symptoms

## Abstract

There is growing interest about the relation between exposure to artificial light at night (LAN) and mental health. This study aims, for the first time, to explore the association between LAN exposure and neuropsychiatric symptoms in individuals with dementia, investigating its role as a potential environmental risk factor. We collected data about 150 patients with dementia in Modena, Italy. We assessed LAN exposure using satellite imagery, while neuropsychiatric symptoms were evaluated through the Neuropsychiatric Inventory (NPI), a caregiver-administered questionnaire measuring the presence and severity of psychiatric symptoms. We used logistic regression models to examine associations between LAN exposure and neuropsychiatric features. Higher LAN exposure was associated with increased risk of psychiatric symptoms, particularly in the domains of psychosis and sleep disturbances. Specifically, individuals exposed to LAN levels above the median (LAN median = 26.32 nW/cm^2^/sr) demonstrated an elevated risk of delusions, hallucinations, and sleep disturbances (OR = 2.09, 95% CI 0.03–4.25), compared to those in the “below the median” category. Associations with other symptom domains, such as apathy and affective disturbances, showed weaker associations. Our findings indicate that artificial nighttime light exposure may negatively impact the mental well-being of individuals with dementia, potentially exacerbating psychotic symptoms and sleep disturbances. However, caution is warranted when interpreting these results due to the limited sample size and the possibility of unexamined residual confounding.

**Supplementary Information:**

The online version contains supplementary material available at 10.1007/s11357-025-01745-z.

## Introduction

The relation between light exposure at night and mental health has received growing attention in recent years, with accumulating evidence indicating that outdoor artificial light at night (LAN) interferes with circadian rhythms [1] and may contribute to various mental disorders [2–5]. Chronic exposure to nighttime light can alter mood, cognition, and metabolic functions, due to disruption in circadian physiology [6]. This interaction extends beyond direct mood effects [7, 8]. Research indicates that sleep disturbances, which are often exacerbated by inappropriate light exposure at night, can disrupt circadian rhythms, which are crucial for maintaining normal sleep–wake cycles, increasing the risk of dementia [9]. Furthermore, chronic nighttime light exposure has been associated with delayed recovery from neurological insults [10] and increased risks of Alzheimer’s disease [11–13]. Conversely, reducing LAN and utilizing light therapy appear to alleviate symptoms and improve outcomes in psychiatric and neurodegenerative diseases [14, 15]. In dementia, the impact of light on neuropsychiatric symptoms is exemplified by the “sundown syndrome” [16], where symptoms worsen in the evening. Nevertheless, the specific relation between outdoor LAN exposure and neuropsychiatric symptoms in dementia remains unexplored. Psychological distress and reduced mental well-being are common symptoms of people with dementia, leading to the development of the so-called behavioral and psychological symptoms or neuropsychiatric symptoms of dementia (BPSD). BPSD include delusions, hallucinations, agitation/aggression, depression, anxiety, euphoria/elation, apathy, disinhibition, irritability, aberrant motor behavior, sleep disturbances, and appetite/eating changes [17]. These symptoms substantially diminish the quality of life for both patients and caregivers while also increasing healthcare costs. Although a range of biological and environmental factors contribute to BPSD, recent evidence suggests that exposure to artificial LAN may be part of the environmental exposome and may act as a potential aggravating factor. As shown in the general population [2] and vulnerable people [18], we hypothesized that LAN exposure could also increase psychiatric symptoms in individuals with dementia. In this study, we aimed to evaluate this association in a cohort of dementia patients from the province of Modena, Northern Italy.

## Methods

### Study population

Newly diagnosed dementia cases were recruited from the Memory Clinics in the province of Modena, Italy, as previously described [19]. The inclusion criteria were as follows: a confirmed dementia diagnosis based on current clinical standards, dementia identified as the main cause of disability, the availability of a caregiver for interviews, residence in the province of Modena, and the availability of the Neuropsychiatry Inventory (NPI), a tool for assessing behavioral symptoms in dementia [17], at the time of recruitment. The recruitment period spanned from October 2016 to February 2020. Exclusion criteria included coexisting diagnoses of pervasive developmental disorder or major psychiatric disorders, as well as cognitive impairment linked to other neurological conditions where disability was primarily due to non-cognitive symptoms (e.g., multiple sclerosis or cerebrovascular disease with severe motor disability). Diagnoses were made according to the clinical diagnosis at the moment of recruitment in Alzheimer’s dementia (AD) [20], vascular dementia (VaD) [21, 22], possible or probable dementia with Lewy body (LBD) [23], frontotemporal dementia (FTD, including behavioral variant, semantic variant of primary progressive aphasia, and non-fluent agrammatic variant of primary progressive aphasia) [24, 25], and atypical parkinsonism (i.e. progressive supranuclear palsy (PSP) or corticobasal degeneration (CBD) [26]). For each patient, we collected, at the time of recruitment, demographic data, clinical information such as type of dementia diagnosis and duration of disease, level of cognitive impairment measured by the Mini Mental State Examination (MMSE [27]), and current address of residence. The study was approved by local Ethical Committee (approval no. 186/2016) and was conducted according to the Declaration of Helsinki. All patients signed a written informed consent.

### Exposure assessment

**Fig. 1:**
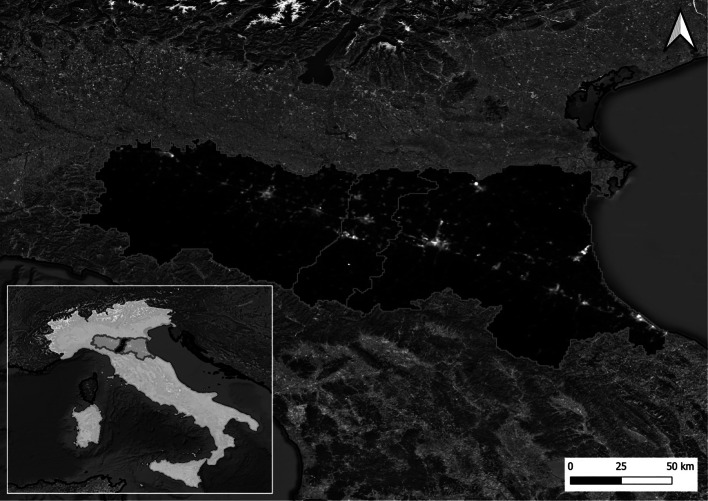
Map of light at night in the Emilia-Romagna region (Northern Italy) with indication of the study area of Emilia-Romagna region and Modena province using 2021 annual mean Visible Infrared Imaging Radiometer Suite (VIIRS) data

We assessed exposure to outdoor artificial LAN at their residence, by geocoding home address at the year of NPI assessment. In line with previous investigation [11], we utilized nighttime light data derived from the Visible Infrared Imaging Radiometer Suite (VIIRS) aboard the Suomi National Polar-Orbiting Partnership (Suomi NPP) spacecraft [28]. The data were obtained from the Global Nighttime Light Maps produced by the Earth Observation Group (EOG) at the Payne Institute of the Colorado School of Mines. The dataset consists of monthly composite radiance images from the VIIRS Day/Night Band (DNB), which are corrected for stray light. This product excludes data impacted by cloud cover, as determined using the VIIRS Cloud Mask (VCM), and does not include data from the edges of the sensor’s scan area. The dataset spans from 2012 to 2024, with a spatial resolution of 15 arc seconds (RS WGS84 latitude/longitude). To assess artificial light exposure for the studied subjects, measured in radiance (nW/cm^2^/sr), we developed a Google Earth Engine procedure that processed imagery from 2014 to 2022. Monthly composites were used to calculate annual averages, from which individual radiance values were extracted for each subject. Figure 1 shows the map of artificial LAN in the Emilia-Romagna region (Northern Italy) with indication of the study area of Emilia-Romagna region and Modena province using 2021 annual mean VIIRS data. Supplementary Figure S1 depicts the map of LAN using annual mean VIIRS data from 2014 to 2022. We used satellite-derived radiance values from the VIIRS-DNB as a proxy for exposure to artificial LAN. These values represent the intensity of upward-directed nighttime illumination measured at the top of the atmosphere, expressed in nanowatts per square centimeter per steradian (nW/cm^2^/sr). Although not directly convertible to luminous flux (lumens), VIIRS-DNB radiance offers a consistent and widely adopted measure of relative LAN exposure across geographic regions. For interpretative context, radiance levels of approximately 20–30 nW/cm^2^/sr typically characterize suburban or semi-urban environments, while values exceeding 50 nW/cm^2^/sr are common in dense urban centers with high-intensity nighttime lighting [29, 30]. While satellite radiance provides spatially resolved, repeatable measurements of nocturnal lighting, the relation between radiance and on-the-ground illumination is influenced by multiple factors, including light source type, emission angle, spectral composition, surface reflectance, and atmospheric conditions. For instance, Bará et al. (2023) demonstrated that a substantial reduction in public lighting in the municipality of Ribeira, Spain—from 93.2 to 28.7 million lumens (Mlm)—resulted in a corresponding drop in VIIRS-DNB radiance from 0.768 to 0.208 Mlm/km^2^ [31]. This supports the utility of satellite data for detecting macro-level changes in lighting, while also highlighting the complexity of interpreting radiance in absolute photometric terms. Our analysis focuses on relative differences in exposure across the study population, which are robust to these limitations.

### Outcome assessment

The presence of behavioral and psychological symptoms was assessed with the NPI, the most frequently used scale to measure neuropsychiatric symptoms in dementia [17]. This semi-structured questionnaire is administered to the caregiver and assesses the presence and severity of 12 symptoms, including delusions, hallucinations, agitation/aggression, dysphoria/depression, anxiety, euphoria/elation, apathy/indifference, disinhibition, irritability/lability, aberrant motor behaviors, sleep behavioral disturbances, and appetite/eating disturbances. The NPI is typically used to assess changes in the patient’s behavior that have appeared in a defined period: for the purpose of the study, we considered changes that occurred in the last months before recruitment. The severity score of each symptom was rated by multiplying severity by frequency; each domain can generate a maximum of 12 points, and thus, the total possible score equals 144 points with higher scores indicating a more severe neuropsychiatric burden. For the purpose of this study, we considered NPI scores resulting from a clustering of symptoms in the following 4 domains [32]: the *Hyperactivity* domain (based on the sum of agitation, disinhibition, irritability, euphoria, and aberrant motor behavior), the *Psychosis* domain (based on the sum of delusions, hallucinations, and sleep behavior disturbances), the *Affective* domain (based on the sum of anxiety and depression), and the *Apathy* domain (based on the sum of apathy and appetite/eating abnormalities). In addition, we conducted separate analyses for sleep disturbances and psychotic symptoms (delusions and hallucinations), to disentangle the effects of LAN on sleep disturbances from those on strictly psychotic symptoms. The clustering of the symptoms (instead of the focus on individual symptoms) is useful because most of the neuropsychiatric symptoms tend to co-occur in clinical practice, and hence, this approach can provide more meaningful interpretations; the clustering of symptoms also helps to avoid the issue of multicollinearity among correlated neuropsychiatric symptoms. For the purpose of this study, the presence of clinically relevant NPS was defined as NPI domain score ≥ 1 (as according to previous studies [33, 34].

### Data analysis

We used a dichotomous classification based on the presence of clinically relevant BPSD as established by NPI score ≥ 1, to define the occurrence and not just the severity of clinically relevant neuropsychiatric symptoms [35, 36]. We used a logistic regression model to calculate odds ratios (ORs) and 95% confidence intervals (95% CI) to estimate the risk of neuropsychiatric symptoms according to different exposure to LAN. The OR was computed considering classification based on LAN median value or tertiles cut points and for continuous 1-unit increase. MMSE values at the time of NPI administration, age, sex, education, disease duration, and dementia diagnosis were included in the multivariable analysis. We also explored the non-linear relation between LAN and neuropsychiatric symptoms risk using a restricted cubic spline regression model with three knots at fixed percentiles (10th, 50th,and 90th). For all estimates, we assessed statistical imprecision through calculation of 95% CI. We used SPSS (Version 24.0, IBM Corp., IBM SPSS Statistics for Windows, Armonk, NY, 2023) and Stata 18.0 (Stata Corp., College Station, TX, 2025) for data analysis.

## Results

A total of 150 eligible patients were included in the present study, 88 females and 62 males. Their characteristics are shown in Table 1. Mean age was 71.0 (± 8.5) years, education was 8.61 (± 4.0) years, disease duration was 69.7 (± 35.8) months, and MMSE score was 16.2 (± 8.5). Median LAN value was 26.32 nW/cm^2^/sr, ranging from 1.03 to 68.56. Diagnosis of AD was made in 91 patients, FTD in 24, VaD in 13, LBD in 8, other atypical parkinsonism in 7, and other dementias (e.g., amyloid angiopathy and normal pressure hydrocephalus) in 7. Neuropsychiatric symptoms were present in 93.3% of patients. Neuropsychiatric symptoms of the *Hyperactivity* domain were present in 74.0% of patients, of the *Psychosis* domain in 36.7% of patients, of the *Affective* domain in 57.3% of patients, and of the *Apathy* domain in 68.0% of patients.

**Table 1:** Clinical and demographical characteristics of patient. AD, Alzheimer’s dementia; FTD, frontotemporal dementia; LAN, light at night; LBD, dementia with Lewy body; MMSE, Mini Mental State Examination; NPI, Neuropsychiatric Inventory; SD, standard deviation; VaD, vascular dementia

	Total *N*= 150
Sex (male, *N*, %)	62 (41.3%)
Age (years, mean, SD)	70.98 (± 8.5)
Education (years, mean, SD)	8.61 (± 4.0)
MMSE (mean, SD)	16.24 (± 8.3)
Disease duration (months, mean, SD)	69.73 (± 35.8)
LAN (nW/cm^2^/sr, median, range)	26.32 (1.03; 68.56)
Diagnosis (*N*, %)	
AD	91 (60.7%)
FTD	24 (16.0%)
VaD	13 (8.6%)
LBD	8 (5.5%)
Atypical parkinsonism	7 (4.6%)
Other	7 (4.6%)
NPI value, median (range)	14.5 (0–57)
NPI total (%)	90.6%
*Hyperactivity* domain	74.0%
*Psychosis* domain	36.7%
*Affective* domain	57.3%
*Apathy* domain	68.0%

**Fig. 2:**
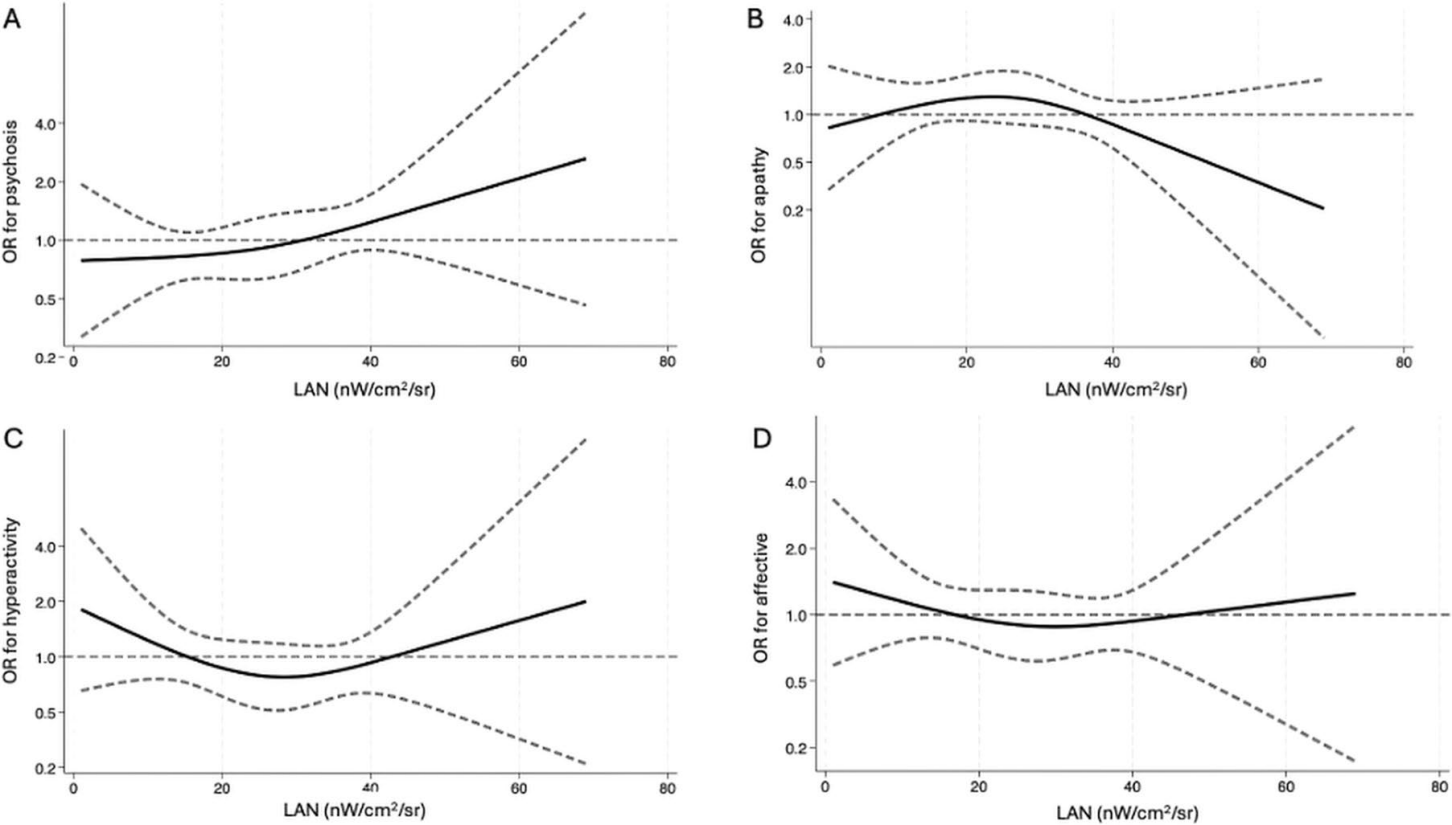
Restricted cubic spline regression analysis for the association between light at night (LAN) values and adjusted odds ratio (OR) of Neuropsychiatric Inventory (NPI) subscales (**A** aOR for psychosis domain; **B** aOR for apathy domain; **C** aOR for hyperactivity domain; **D** aOR for affective domain). The solid black line represents the risk estimate adjusted for MMSE, age, sex, education, disease duration, and dementia diagnosis, and the dotted lines represent the 95% confidence interval

**Fig. 3:**
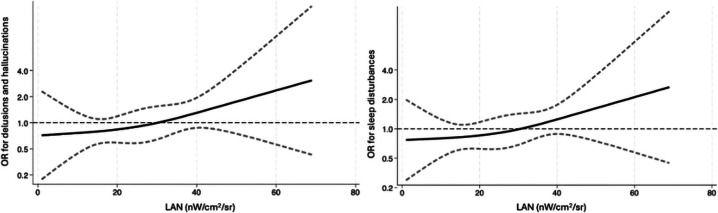
Restricted cubic spline regression analysis for the association between artificial light at night (LAN) values and adjusted odds ratio (aOR) of delusions/hallucinations (on the left) and sleep disturbances (on the right) subscales. The solid black line represents the risk estimate adjusted for MMSE, age, sex, education, disease duration and the dotted lines represent the 95% confidence interval

Table 2 reports adjusted neuropsychiatric symptoms domains OR (and 95%CI), according to LAN values. We observed that higher level of LAN was associated with greater risk of neuropsychiatric symptoms in the psychosis domain (for LAN above the median value, OR = 2.09, 95% CI 0.03–4.25), with a stronger association for higher LAN values (for LAN > 34 nW/cm^2^/sr, OR = 2.51, 95% CI 1.06–5.95). The spline regression analysis depicted a non-linear pattern with risk of symptoms of the psychosis domain rapidly increasing at higher LAN values, particularly at the upper part of the exposure range (Fig. 2A). As for the apathy domain, the association between risk and LAN showed a variable pattern. More precisely, for one-unit increase of LAN and when considering LAN median value as cutoff, we found no substantial associations with apathy. Conversely, when measuring LAN exposure with tertiles while considering the lowest tertile as the reference, we found that the intermediate tertile was associated with higher risk of apathy, whereas the highest tertile was associated with lower risk of apathy. Spline regression analysis showed a more pronounced inverse association between LAN values and apathy risk mainly for higher LAN values, whereas lower LAN values were associated to lower apathy risk (Fig. 2B). Concerning the affective and hyperactivity domains, we did not find substantial association with LAN exposure. The spline regressions analysis showed a possible unfavorable effect on affective and hyperactivity symptoms by both highest and lowest LAN levels (Fig. 2C and D). The separate analyses for sleep disturbances and strictly psychotic symptoms (Table 3 and Fig. 3) confirmed that higher LAN values are associated to higher risk of both psychotic symptoms (i.e., delusion and hallucinations) and sleep disturbances (for LAN above median, OR of psychotic symptoms = 2.97, 95% CI 1.18–7.50; for highest LAN tertile, OR of psychotic symptoms = 3.25, 95% CI 1.12–9.39; for highest LAN tertile, OR of sleep disturbances = 2.93, 95% CI 1.16–7.38).

**Table 2:** Adjusted odds ratio (aOR) for neuropsychiatric symptoms assessed through Neuropsychiatric Inventory (NPI) domains based on light at night (LAN) values. Mini Mental State Examination, education, age, sex, disease duration, and diagnosis were entered in the analyses for adjustment. CI, confidence interval

	NPI hyperactivity Domain ≥ 1 (*N*= 111)		NPI psychosis Domain ≥ 1 (*N*= 55)		NPI apathy Domain ≥ 1 (*N*= 102)		NPI affective Domain ≥ 1 (*N*= 86)	
	aOR	95% CI	aOR	95% CI	aOR	95% CI	aOR	95% C
LAN above the median	1.06	0.49–2.28	2.09	1.03–4.25	1.11	0.54–2.28	0.95	0.48–1.86
LAN 1-unit increase	0.99	0.97–1.02	1.02	0.99–1.05	0.98	0.97–1.01	0.99	0.97–1.01
LAN tertiles								
1 st tertile (< 18) (reference)	-	-	-	-	-	-	-	-
2nd tertile (18–33)	0.71	0.27–1.83	0.98	0.39–2.42	1.25	0.50–3.09	0.44	0.19–1.05
3rd tertile (> 34)	0.84	0.31–2.21	2.51	1.06–5.95	0.93	0.38–2.29	1.02	0.43–2.40

**Table 3:** Adjusted odds ratio (aOR) for delusion/hallucinations and sleep disturbances Neuropsychiatric Inventory (NPI) domains based on light at night (LAN) values. Mini Mental State Examination, education, age, sex, disease duration, and diagnosis were entered in the analyses for adjustment. CI, confidence interval

	NPI delusion/hallucination ≥ 1 (*N*= 29)		NPI sleep disturbances ≥ 1 (*N*= 41)	
	aOR	95% CI	aOR	95% CI
LAN above the median	2.97	1.18–7.50	1.90	0.89–4.06
LAN 1-unit increase	1.01	0.99–1.05	1.01	0.99–1.04
**LAN tertiles**				
1 st tertile (< 18) (reference)	-	-	-	-
2nd tertile (18–33)	0.83	0.24–2.90	1.07	0.40–2.86
3rd tertile (> 34)	3.25	1.12–9.39	2.93	1.16–.7.38

## Discussion

In this study, we tested the hypothesis that higher exposure to LAN may negatively impact mental well-being in individuals with dementia. We specifically focused on neuropsychiatric symptoms, which are among the most common manifestations of psychological distress and reduced mental well-being in cognitively impaired populations. These symptoms are well-established contributors to greater functional decline, reduced quality of life, increased caregiver burden, and elevated healthcare costs [37, 38]. As expected, given the moderate severity of dementia in our sample (mean MMSE: 16.2), we observed a high prevalence of neuropsychiatric symptoms. Notably, the association with LAN exposure varied across symptom domains. High LAN exposure was strongly associated with an increased risk of psychotic symptoms and sleep disturbances, even after adjusting for key clinical covariates including age, sex, cognitive status, disease duration, and dementia subtype. Associations with other neuropsychiatric symptoms were more variable and generally weaker.

Consistent with our findings that increasing LAN exposure is associated with a higher risk of psychotic symptoms, previous research in individuals with pre-existing mental health conditions has linked nighttime exposure to artificial light with the worsening of psychotic symptoms [4, 5, 18]. Indeed, disruptions in circadian rhythms caused by environmental factors, including light exposure, have been associated with the onset and worsening of psychotic symptoms, such as delusions and hallucinations, particularly in individuals experiencing a first episode of psychosis [39]. Furthermore, exposure to brighter nighttime light has been associated with an increased risk of manic or hypomanic episodes in people with bipolar disorder [40]. Hypersensitivity of the circadian system to nocturnal light, reduced amplitude of behavioral rhythms, and greater circadian variability have also been proposed as trait markers of bipolar disorder [41, 42]. Supporting this, interventions such as dark therapy and the use of blue-light-blocking glasses have demonstrated efficacy in alleviating manic symptoms [43, 44]. In parallel, pharmacological treatments for bipolar disorder have been found to reduce the circadian system’s sensitivity to light [45], further emphasizing the therapeutic potential of modulating light exposure. In line with this body of evidence, our findings suggest that LAN may act as an environmental risk factor for the exacerbation of psychotic symptoms even in people with dementia, potentially via circadian disruption. The observed association between high exposure to LAN and increased risk of psychotic symptoms, particularly delusions and hallucinations, suggests a clinically meaningful effect beyond any consideration related to nominal statistical significance. Neuropsychiatric symptoms are known to substantially worsen prognosis in dementia, being associated with faster cognitive decline, earlier institutionalization, and heightened caregiver burden [46]. Given the limited efficacy and considerable risks associated with antipsychotic medications in this population [47], identifying modifiable environmental risk factors such as LAN becomes especially relevant. Clinically, our findings support the incorporation of environmental light assessments into routine dementia care. Non-pharmacological interventions aimed at improving light hygiene—such as reducing nighttime ambient light, using blackout curtains, or optimizing indoor light exposure according to circadian rhythms—could be considered in management strategies for BPSD. These approaches may be particularly useful in urban settings where artificial light exposure is widespread and difficult to avoid. On a broader level, our results raise considerations for public health and urban planning. As aging populations increasingly reside in urbanized environments, nighttime lighting policies should consider their potential impact on neuropsychiatric health. Prior studies have linked LAN to circadian disruption, depressive symptoms, and impaired sleep regulation in older adults [2, 11]. Our findings add to this body of evidence, highlighting the need for multidisciplinary strategies—including urban design and residential zoning—to decrease unnecessary nighttime light exposure as part of dementia-friendly community planning, as suggested by recently proposed urban guidelines for human well-being [48].

Regarding sleep disturbances, our results indicate that higher levels of LAN exposure are associated with an increased risk of sleep-related symptoms. This finding aligns with previous literature linking LAN to disruption in circadian rhythms and mental health, particularly through its effect on sleep–wake cycles, melatonin secretion, and metabolic processes, as shown in both animal [49] and human studies [50–52]. Circadian rhythms regulate multiple biological processes relevant to depression and mood disorders—including brain plasticity, neurotransmission, hormonal balance, and clock gene expression—making these systems especially vulnerable to environmental disturbances such as nighttime light exposure [53, 54].

When exploring the relationship between LAN exposure and apathy, we observed a variable and weak effect of LAN: lower LAN levels appear to be associated with an increased risk of apathy, while higher LAN levels seem to be linked to a decreased risk. This is in line with clinical findings proposing that targeted light exposure can have therapeutic effects on mood and cognitive engagement, potentially mitigating apathy, as demonstrated in a recent study in individuals with Korsakoff Syndrome, where light intervention led to a notable decrease in apathy [55]. Apathy in dementia is thought to be linked to disruptions in dopaminergic frontostriatal circuits as well as to circadian dysfunction. We may speculate that the moderate levels of LAN may be associated with higher behavioral engagement and increased dopaminergic activity via greater arousal and motivation, thereby mitigating apathetic behaviors [9]. Additionally, light exposure is known to acutely suppress melatonin and increase alertness, thus increasing motivation and initiative [9]. Additionally, light exposure is known to acutely suppress melatonin and increase alertness, potentially influencing motivational circuits [10]. Nonetheless, this finding must be interpreted with caution, due to the relatively limited sample size and the potential effect of multiple comparisons. Although our primary models were pre-specified and adjusted for key covariates, we acknowledge that this association should be regarded as hypothesis-generating. Furthermore, confidence intervals of the risk estimates highlighted their statistical imprecision. Taken together, these considerations highlight the need for replication in larger, independent cohorts before any definitive conclusions can be drawn regarding this potential relation.

We found more variable and weaker associations between LAN and the other neuropsychiatric symptoms. More precisely, symptoms in the affective and hyperactivity domain were associated with either higher or, to a lesser extent, lower LAN exposure values, suggesting that “extreme” (i.e., highest or lowest) LAN levels may have a possible detrimental effect on these neuropsychiatric symptoms, whereas “intermediate” LAN values may have a “protective” effect.

This study has important limitations: first, we did not collect measures of indoor light exposure, which may be different from the outdoor LAN exposure that we measured. Nevertheless, recent studies have shown that residing in areas with higher levels of outdoor LAN is linked to shorter sleep duration, increased daytime sleepiness, and dissatisfaction, meaning that outdoor LAN alone can significantly influence biological processes [58]. Another limitation of our study is the small sample size, which compromises the statistical precision of the risk estimates; this requires caution in interpreting the results and emphasizes the need for further investigation in larger-scale studies. In addition, the cross-sectional design of the study precludes causal inference between LAN exposure and neuropsychiatric symptoms, underscoring the need for longitudinal studies to better elucidate the directionality and underlying mechanisms of these associations. No formal power calculation was conducted for this study, as it is based on a secondary analysis of data from a population-based observational cohort described in Chiari et al. (2021) [19]. The sample size was determined by the number of participants meeting inclusion criteria within the original study, thus limiting the precision of some estimates. Lastly, we did not collect data on ambient air pollution or noise, both of which are recognized as potential contributors to dementia risk [11] and neuropsychiatric symptoms [59, 60]. Future research employing multivariable modeling to investigate the combined effects of the totality of environmental exposome on neuropsychiatric symptoms could provide valuable insights into the role of all environmental factors in dementia patients.

Despite these limitations, our study suggested for the first time that, alongside various biological and environmental factors known to contribute to neuropsychiatric symptoms in dementia, exposure to artificial LAN might also exacerbate mental distress and psychotic symptoms.

## Supplementary Information

 Below is the link to the electronic supplementary material.

ESM 1 (DOCX 225 KB)
