# Supplementary material for: Outdoor light at night and neuropsychiatric symptoms in dementia
Source: GeroScience. 2025 Jul 15;48(2):2389–99. doi: 10.1007/s11357-025-01745-z (PMC12972467; doi:10.1007/s11357-025-01745-z)

**Supplementary Figure S1.** Map of light at night in the Emilia-Romagna region (Northern Italy) with indication of the study area of Emilia-Romagna region and Modena province using annual mean Visible Infrared Imaging Radiometer Suite (VIIRS) data from 2014 to 2022.


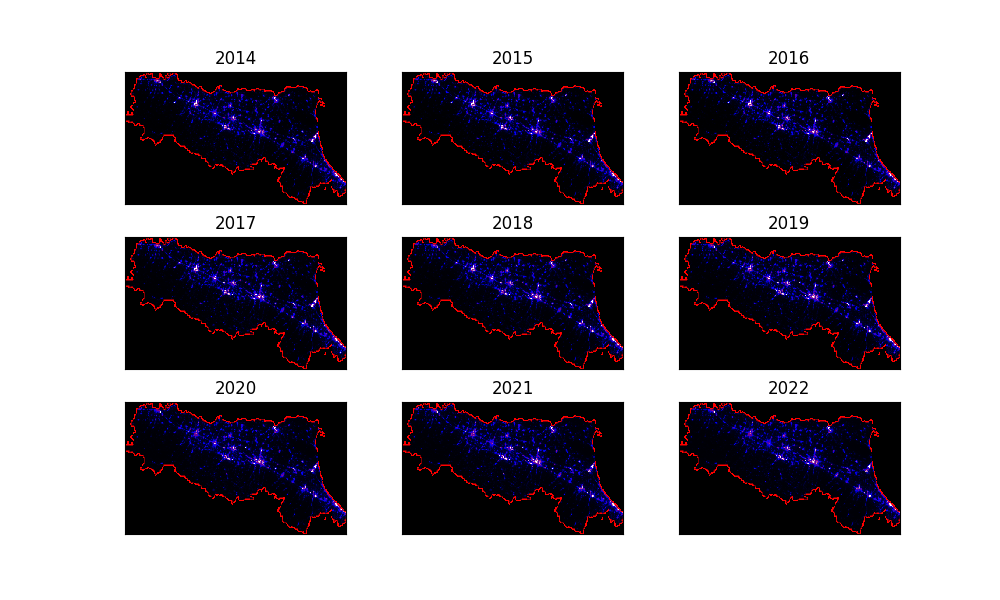

Supplement: Supplementary file 1 — (DOCX 225 KB) [file 11357_2025_1745_MOESM1_ESM.docx]
